# Risk of first recurrence after treatment in a population-based cohort of young women with breast cancer

**DOI:** 10.1007/s10549-024-07338-2

**Published:** 2024-04-30

**Authors:** Robin Schaffar, Simone Benhamou, Pierre O. Chappuis, Elisabetta Rapiti

**Affiliations:** 1 Geneva Cancer Registry, Global Health Institute, University of Geneva, Geneva, Switzerland; 2 INSERM Unit 1018, Research Centre on Epidemiology and Population Health, Villejuif, Île-de-France, France; 3 Division of Precision Oncology, Geneva University Hospitals, Geneva, Switzerland; 4 Division of Genetic Medicine, Geneva University Hospitals, Geneva, Switzerland

**Keywords:** Breast cancer, Recurrences, Young women, Population-based studies

## Abstract

**Purpose:**

Breast cancer (BC) in women under 45 is rare yet often aggressive. We aim to analyze loco-regional recurrences (LR), distant recurrences (DR), second breast cancers, and mortality in young BC patients.

**Methods:**

We enrolled 776 women with non-metastatic BC ≤45 years diagnosed from 1970 to 2012. Variables included age, family history, tumor stage/grade, and treatment. We used multivariate Cox regression and competing risk models.

**Results:**

Among the participants, 37.0% were diagnosed before the age of 40. Most had stage I or II, grade II, ER- and PR-positive, HER2-negative tumors. Over a median follow-up of 8.7 years, 10.1% experienced LR, 13.7% developed DR, and 10.8% died, primarily due to BC. The majority of recurrences occurred within the first five years. Older age (>40) significantly reduced the risk of LR and DR. Advanced disease stage, certain surgical strategies, and positive margins increased DR risk. In the cohort diagnosed between 2001 and 2012, recent diagnosis, triple-negative cancer, and hormonal therapy were associated with reduced LR risk. Breast-conserving surgery appeared to offer protective effects against DR.

**Conclusion:**

This study highlights that BC in young women carries a significant risk of early recurrence, with age, tumor characteristics, and treatment modalities influencing outcomes. The findings emphasize the need for tailored treatment strategies for young BC patients, focusing on surgical precision and aggressive adjuvant therapy for high-risk cases. This research contributes valuable insights into managing BC in younger patients, aiding in improving long-term outcomes.

**Supplementary Information:**

The online version contains supplementary material available at 10.1007/s10549-024-07338-2.

## Background

A breast cancer (BC) diagnosis in young women is relatively rare. In 2020, the age-standardized incidence rate in Europe for women aged 45 and older was estimated at 228.6 per 100,000, compared to 20.1 per 100,000 for those 45 years or younger [1]. Breast cancer is the leading cause of cancer death among women under 45 [1].

Young women with BC typically present with larger, more aggressive, and advanced tumors, leading to shorter survival [2]. They have a higher risk of developing recurrences [3–5]. Particularly, loco-regional recurrences (LR) in this group have been linked to increased risks of distant metastasis and mortality [6]. BC subtypes also influence recurrence patterns. HER2-positive and triple-negative tumors, more common in younger women, are prone to local and distant recurrences (DR) [2, 7].

The risk of loco-regional and distant recurrences for young women with breast cancer has not been extensively investigated. Young patients with breast cancer usually represent a very small subset of eligible patients in randomized clinical trials and observational study populations, the latter often reporting only on hospital-selected patients [8]. Few population-based studies have specifically addressed this demographic [7, 9, 10].

A study by Aalders et al. noted a decline in 5-year local and regional recurrence rates in patients under 35 from 2003 to 2008, attributing this improvement to advancements in treatment and management [11]. The role of systemic therapy and targeted drugs in reducing recurrence risk is acknowledged, but longer follow-up is needed for accurate risk assessment in very young women.

Furthermore, when estimating the absolute risk of recurrence and death for clinical purposes or risk stratification, it is important to consider that these events are not independent from each other and that the estimation without considering competing risks could be biased. In general, it tends to overestimate the risk and the bias increases with length of follow-up and for less common end-points as local recurrences (LR) [12].

We constituted a retrospective population-based cohort of breast cancer women aged ≤45 years in Geneva, Switzerland, with the purpose of evaluating tumor characteristics, patterns of care, prognostic factors, and outcomes of breast cancer in this population [13]. In the present article, we use competing risk analysis to describe the long-term patterns of loco-regional recurrences, distant recurrences, second breast cancer, and death in this cohort of young women. This approach allows us to avoid biased risk estimates. In addition, we investigated factors associated with the development of recurrences.

## Methods

The cohort has been enrolled using the population-based Geneva Cancer Registry (GCR), which records since 1970 all incident cancers occurring in the population of the county (approximately 500,000 inhabitants in 2023). The detail study protocol has been previously described [13]. In brief, all women resident in the canton of Geneva and diagnosed with non-metastatic primary invasive breast cancer (stages I–III) at the age of 45 years or less between 1970 and 2012 were enrolled in the cohort. Women with previous or synchronous invasive cancer were not included.

Data routinely registered from the GCR include patient, tumor, and treatment characteristics. Pathological and medical files were reviewed to collect additional data, such as family history of breast cancer and recurrences. As estrogen and progesterone receptor (ER and PR) status recording began in 1995, the study was limited to patients diagnosed from 1995 to 2012 who underwent surgical treatment.

Individual characteristics of interest were age at diagnosis, year of diagnosis, and family history of breast and/or ovarian cancer. Familial risk was categorized as high (at least one first-degree relative with breast/ovarian cancer diagnosed before the age of 50 years, or at least two first-degree relatives with breast/ovarian cancer at any age, or at least three cases of breast/ovarian cancer among first- or second-degree relatives), low (no affected first- or second-degree relatives with breast/ovarian cancer), or moderate (all other known family histories) according to a previous study by our group [14]. Tumor variables considered were tumor stage and tumor differentiation coded according to the TNM using the pathological classification and when missing the clinical classification [15]. ER and PR status were categorized as positive (when at least 1% of the receptors were positive), negative, or unknown. The information about the Ki67 and HER2 status were collected only since 2000 and 2001, respectively. Therefore, we were able to classify tumor biology as expressed by tumor biomarker subtypes only for the subset of women diagnosed since 2001. Based on ER and PR status, Ki67, expression of HER2 and grade, four breast cancer surrogate subtypes were defined [16, 17]: Luminal A-like: (Ki67 <14%, or if unknown grade I or II, HER2−, ER+, and/or PR+); Luminal B-like (any Ki67 or any grade, HER2+, ER+, and/or PR+; Ki67 ≥14%, or if unknown grade III, HER2−, ER+, and/or PR+); HER2+/HR− (HER2+, ER−, and PR−); and Triple negative (HER2−, ER−, and PR−). The surgical treatments under consideration included breast-conserving surgery with radiotherapy, mastectomy without radiotherapy, mastectomy with radiotherapy, and other combination of surgical procedures. Additionally, we assessed the administration of chemotherapy (Yes/No) and hormonal treatment (Yes/No).

Women were followed for loco-regional and distant recurrences, second breast cancer occurrence or death up to 31/12/2015. The information about LR and DR were collected from the clinical files of the patients. LR was defined as any breast cancer in surgical scar, in skin and subcutaneous tissue on the ipsilateral breast and thoracic wall if the morphology was similar as the primary tumor, in ipsilateral axillary, infraclavicular, supraclavicular, internal mammary/parasternal, or intramammary lymph node. DR was defined as breast cancer in any organ other than the breast, excluding the events listed under LR. Information about the second breast cancer was collected from the GCR. Second breast cancer was defined as any epithelial breast cancer, in situ or invasive, in the contralateral breast, or in the ipsilateral breast if of different morphologies from the first one [18]. Vital status was ascertained through passive (hospital and mortality certificates) and active follow-up using the files of the Cantonal Population Office in charge of registration of the resident population of the Canton.

Cumulative incidence was estimated for LR and DR in a competing risks framework which considered second breast cancer and deaths from all causes as competing events. Analyses were further stratified by age, stage, grade, and ER status.

To investigate factors associated with the occurrence of LR and DR in the presence of competing risk events, including second cancer occurrences and mortality, we conducted multivariate Cox regression analyses on a complete case basis. This method involved only participants with complete data on all variables of interest. Our analyses considered a predefined set of covariates selected a priori based on their established prognostic impact. These analyses were applied to two distinct cohorts: the whole cohort, with all the cases diagnosed between 1995 and 2012, and the cohort of women diagnosed 2001 and 2012 for whom it was available the surrogate molecular subtype. The covariates, chosen based on prior knowledge of their relevance, were age at diagnosis, year of incidence (continuous), family history, disease stage, grade, ER status (for the 1995–2012 cohort) or surrogate molecular type (for the 2001–2012 cohort), surgical treatment strategy, surgical margins, and the administration of chemotherapy and hormonal treatment.

Given that we are operating within a competing risk setting, it is important to note that these LR and DR models are inherently interconnected, as understanding the risk factors for one event may provide insights into the occurrence of the other event.

All the results were considered statistically significant at a p value < 0.05. All the analyses were performed using STATA software (version 17, [19]).

## Results

A total of 776 young women with breast cancer were included in the study. Patient, tumor, and treatment characteristics of the women in this cohort are shown according to the type of first event experienced (Table 1).

**Table 1:** Individual characteristics (a), tumors characteristics (b), and treatment strategies (c) of young women with breast cancer according to outcome. Geneva 1995–2012

	First event (mutually exclusive)											
	Total		No event		Local recurrence		Distant recurrence		2nd breast cancer		Death	
	N	%	N	%	N	%	N	%	N	%	N	%
*(a)*												
Age class												
<40 years	287	37	199	33.9	33	47.1	38	50	14	43.8	3	27.3
40–45 years	489	63	388	66.1	37	52.9	38	50	18	56.3	8	72.7
Period of diagnosis												
1995–2000	210	27.1	131	22.3	26	37.1	34	44.7	14	43.8	5	45.5
2001–2006	292	37.6	219	37.3	35	50	21	27.6	14	43.8	3	27.3
2007–2012	274	35.3	237	40.4	9	12.9	21	27.6	4	12.5	3	27.3
Family history of breast/ovarian cancer												
No	476	63.5	356	63.1	45	66.2	50	66.7	18	56.3	7	63.6
Yes, moderate	210	28	155	27.5	21	30.9	23	30.7	8	25	3	27.3
Yes, high	64	8.5	53	9.4	2	2.9	2	2.7	6	18.8	1	9.1
Missing	26		23		2		1		0		0	
*(b)*												
Stage												
Stage I	301	39.8	242	41.9	28	43.1	16	22.2	12	38.7	3	27.3
Stage II	362	47.8	272	47.1	30	46.2	40	55.6	13	41.9	7	63.6
Stage III	94	12.4	64	11.1	7	10.8	16	22.2	6	19.4	1	9.1
Missing	19		9		5		4		1		0	
Estrogen receptors												
Negative	172	22.7	117	20.3	17	25.4	23	31.1	12	37.5	3	30
Positive	586	77.3	458	79.7	50	74.6	51	68.9	20	62.5	7	70
Missing	18		12		3		2		0		1	
Progesterone receptors												
Negative	230	29.6	166	28.3	22	31.4	28	36.8	11	34.4	3	27.3
Positive	528	68.0	409	69.7	45	64.3	46	60.5	21	65.6	7	63.6
Missing	18		12		3		2		0		1	
Grade												
I	144	19	111	19.3	14	20.6	12	16.2	7	22.6	0	0
II	365	48.1	284	49.3	30	44.1	33	44.6	13	41.9	5	50
III	250	32.9	181	31.4	24	35.3	29	39.2	11	35.5	5	50
Missing	17		11		2		2		1		1	
Available since 2001												
HER2 status												
Positive	119	23.6	93	22.80	9	25.7	12	29.3	3	18.8	2	40.0
Negative	386	76.4	315	77.20	26	74.3	29	70.7	13	81.3	3	60.0
Missing	61		48		9		1		2		1	
Surrogate molecular subtype												
Luminal A-like	200	39.8	174	42.8	13	37.1	8	19.5	5	33.3	0	0
Luminal B-like	212	42.1	166	40.8	17	48.6	20	48.8	6	40	3	60
HER2+/HR−	31	6.2	24	5.9	1	2.9	4	9.8	2	13.3	0	0
Triple negative	60	11.9	43	10.6	4	11.4	9	22	2	13.3	2	40
Missing	63		49		9		1		3		1	
*(c)*												
Surgical treatment strategy												
BCS + RT	461	59.4	362	54.3	38	46.1	35	46.1	21	65.6	5	45.5
Mastectomy alone	124	16.0	95	16.2	13	18.6	12	15.8	2	6.3	2	18.2
Mastectomy + RT	140	18.0	90	15.3	12	17.1	29	38.2	7	21.9	2	18.2
Others	51	6.6	40	6.8	7	10.0	0	0.0	2	6.25	2	18.18
Surgical margins												
Negative	640	87.8	499	89.9	52	81.3	58	82.9	24	82.8	7	63.6
Borderline	56	7.7	40	7.2	6	9.4	4	5.7	4	13.8	2	18.2
Positive	33	4.5	16	2.9	6	9.4	8	11.4	1	3.4	2	18.2
Missing	19		11									
Chemotherapy												
No	182	23.7	138	23.8	21	30.0	10	13.2	10	31.3	3	27.3
Yes	586	76.3	441	76.2	49	70.0	66	86.8	22	68.8	8	72.7
Missing	8		8		0		0		0		0	
Hormonal treatment												
No	241	31.8	165	28.9	24	34.3	29	39.2	17	53.1	6	54.5
Yes	516	68.2	405	71.1	46	65.7	45	60.8	15	46.9	5	45.5
Missing	19		17		0		2		0		0	

Breast cancer was diagnosed before the age of 40 years in 287 women (37.0%) and between 40 and 45 years in 489 women (63.0%). Most women had no family history of breast and/or ovarian cancer (63.5%). Most tumors were diagnosed at stage I (*n*=301, 39.8%) or II (*n*=362, 47.8%) and of grade II (*n*=365, 48.1%), were ER-positive and PR-positive (*n*=586, 77.3% and *n*=528, 68.0%, respectively), HER2-negative (*n*=386, 76.4%), and of luminal A-like or luminal B-like molecular subtype (*n*=200, 39.8% and *n*=201, 42.1%, respectively). Most women were treated with chemotherapy (*n*=586, 76.3%) and hormonotherapy (*n*=516, 68.2%) and had breast-conserving surgery (BCS) as surgical strategy (*n*=489, 64.2%).

At the end of the follow-up (median: 8.7 years), most women (*n*=587, 75.64%) did not experience any of the study events; 78 women (10.1%) had at least one LR, 106 (13.7%) developed a DR, 36 (4.6%) were diagnosed with a second primary breast cancer, and 84 women (10.8%) died during the study period, 68 of whom from BC. The median time to the first event was 7.3 years.

**Fig. 1:**
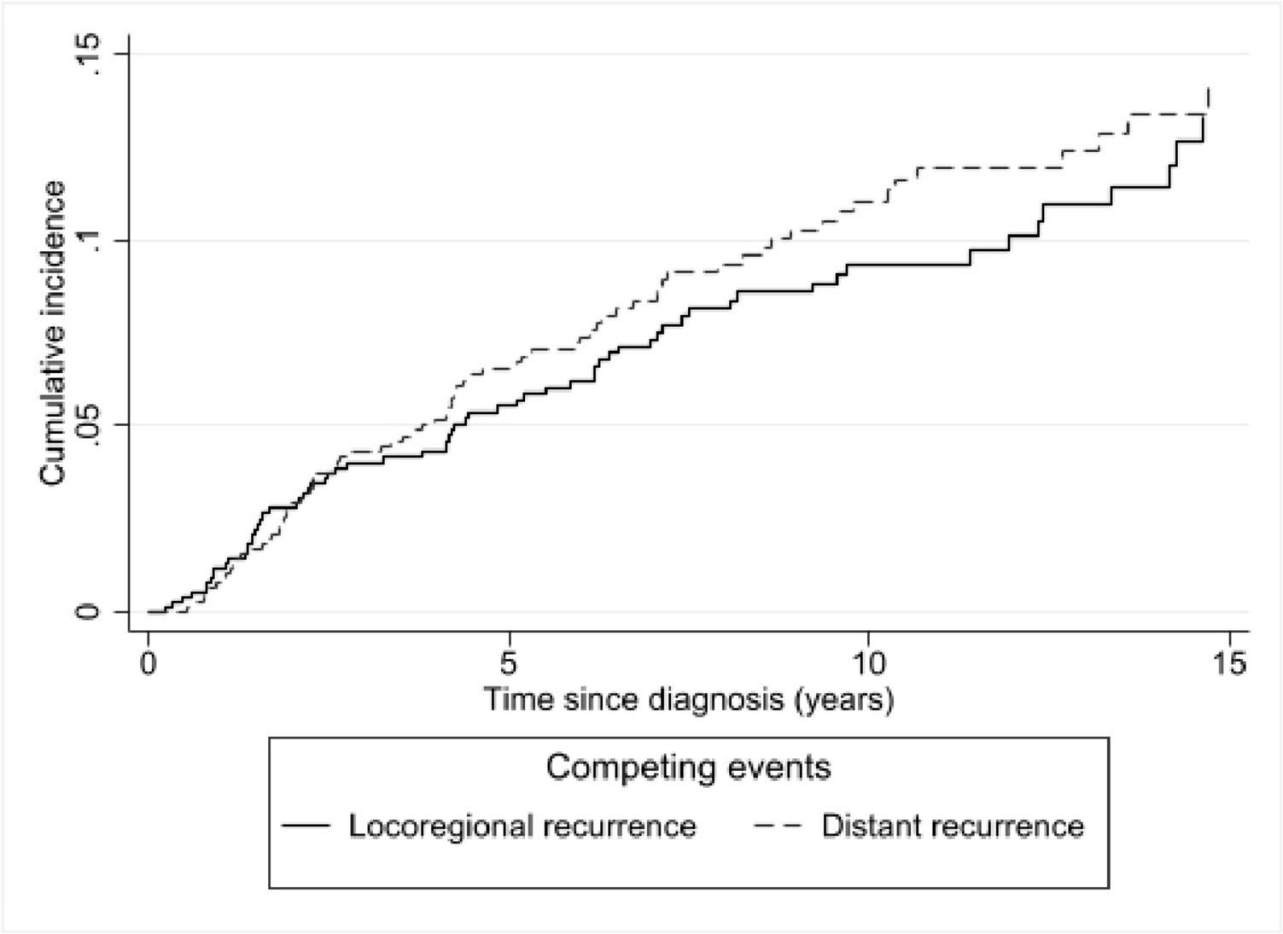
Cumulative incidence of loco-regional recurrence and distant recurrence under the competing risk model in young women with breast cancer. Geneva, 1995–2012

The competing risk analysis considering the occurrence of the first event in presence of competing risk events showed that recurrences occurred throughout the follow-up period. After 5, 10, and 15 years of follow-up, the cumulative incidence of LR was 5.5% [95% CI: 3.9–7.2], 9.4% [95% CI: 7.1–11.8], and 13.3% [95% CI: 10.0–17.1] that of DR was 6.5% [95% CI: 4.8–8.4], 11.1% [95% CI: 8.4–13.5], and 14.1% [95% CI: 10.4–17.1]. (Fig. 1). Most of the recurrences occurred during the first 5 years of follow-up (57% for LR and 62% for DR, respectively).

The cumulative incidence rates for LR were very similar between women aged less than 40 and 40–45 during the first two years after diagnosis and then younger women showed higher rates although never statistically different from women 40 to 45 years old (Suppl. Fig. 1a). For DR, already one year after diagnosis, younger women showed higher rates, and at 5 years, their cumulative incidence was 9. 3% [95% CI: 6.1–13.2] vs. 5.0% [95% CI: 3.3–7.3] for women 40–45 years old (Suppl. Fig. 1b).

No statistical differences were found for LR according to stage at diagnosis during the first 5 years of follow-up, although a trend toward higher incidence rates with higher stage was observed (Suppl. Fig. 2a). For DR, stage III tumors had a cumulative incidence rate always higher than tumors with lower stages at diagnosis, at 5 years being 15.1% (95% CI: 8.5–23.5) compared to 2.9% (95% CI: 1.4–5.5) for stage I tumors (Suppl. Fig. 2b).

The cumulative incidence of LR according to grade at diagnosis showed clear differences, with grade I tumors presenting very low rates of recurrence up to 5 years (0.7%, 95% CI: 1–3.7), while grade III tumors presented a rate of almost 8% (95% CI: 4.9–11.8) and grade II tumors rates were in between the two (Suppl. Fig. 3a). On the contrary, for DR, although a gradient with increasing grade was seen, the differences were not statistically significant (Suppl. Fig. 3b).

The cumulative incidence according to ER status showed a higher loco-regional rate in the first 5 years of follow-up among women ER-negative compared to women with ER-positive tumors, although not statistically significant. (Suppl. Fig. 4a). The cumulative incidence of DR increased steeply during the first years after diagnosis for ER-negative tumors (11.7% at 5 years, 95% CI: 7.3–17.2) compared with ER-positive tumors (4.9%, 95% CI: 3.3–6.9). (Suppl. Fig. 4b).

In the multivariate Cox analysis of the whole cohort of women diagnosed between 1995 and 2012, the only factor that showed a significant association with LR development as first event was age. Notably, women aged 40 and older presented a reduced hazard ratio (HR) of 0.51 (95% CI: 0.30–0.87) compared to women who were <40 years old at diagnosis. Similarly, older women showed a significant decreased risk of developing DR as first event (HR: 0.47, 95% CI: 0.28–0.78). Other factors associated with DR were a more advanced stage (HR of stage III vs stage I: 3.06, 95% CI: 1.25–7.52), surgical strategy (HR of mastectomy plus RT vs BCS plus RT: 2.21, 95% CI: 1.18–4.14), and positive margins after surgery (HR: 2.88, 95%CI: 1.28–6.49) (Table 2).

**Table 2:** Multivariate Cox regressions modeling the risk of loco-regional and distant recurrences in a cohort of young women with breast cancer according to the period of diagnosis. Geneva 1995–2012 and 2001–2012

	Cohort 1995–2012						Cohort 2001–2012					
	Local relapse			Distant relapse			Local relapse			Distant relapse		
	HR	95% CI	*p*-value	HR	95% CI	*p*-value	HR	95% CI	*p*-value	HR	95% CI	*p*-value
Age class												
<40	**1**			**1**			**1**			**1**		
40+	**0.51**	[0.3–0.87]	0.014	**0.47**	[0.28–0.78]	0.004	**0.66**	[0.31–1.39]	0.275	**0.41**	[0.2–0.83]	0.013
Year of incidence	**0.96**	[0.9–1.03]	0.232	**1.00**	[0.94–1.06]	0.897	**0.83**	[0.72–0.96]	0.010	**1.03**	[0.92–1.16]	0.596
Family history												
None	**1**			**1**			**1**			**1**		
Yes, moderate	**1.32**	[0.75–2.31]	0.34	**1.19**	[0.7–2.03]	0.526	**0.98**	[0.45–2.13]	0.952	**1.57**	[0.79–3.14]	0.200
Yes, high	**0.43**	[0.1–1.84]	0.258	**0.22**	[0.03–1.66]	0.143	**0.41**	[0.05–3.2]	0.393	**0.33**	[0.04–2.58]	0.290
Stage												
I	**1**			**1**			**1**			**1**		
II	**0.95**	[0.51–1.74]	0.857	**1.63**	[0.83–3.2]	0.154	**1.40**	[0.6–3.27]	0.433	**1.94**	[0.75–5.01]	0.169
III	**1.44**	[0.49–4.21]	0.506	**3.06**	[1.25–7.52]	0.014	**0.84**	[0.15–4.74]	0.846	**4.99**	[1.51–16.5]	0.008
Grade												
Well differentiated	**1**			**1**			**1**			**1**		
Moderately diff.	**1.14**	[0.55–2.35]	0.727	**0.75**	[0.37–1.51]	0.415	**1.55**	[0.5–4.86]	0.448	**0.48**	[0.17–1.41]	0.184
Poorly/Not diff.	**1.44**	[0.64–3.23]	0.375	**0.97**	[0.45–2.08]	0.943	**2.41**	[0.62–9.36]	0.205	**0.57**	[0.18–1.84]	0.348
ER												
Negative	**1**			**1**			–	–	–	–	–	–
Positive	**0.83**	[0.35–1.93]	0.659	**0.84**	[0.37–1.93]	0.687	–	–	–	–	–	–
Histological subtype												
Luminal A	–	–	–	–	–	–	**1**			**1**		
Luminal B	–	–	–	–	–	–	**0.82**	[0.34–1.96]	0.652	**3.17**	[1.18–8.55]	0.022
Her2 enriched	–	–	–	–	–	–	**0.15**	[0.01–1.54]	0.110	**3.21**	[0.49–21.05]	0.223
Triple neg.	–	–	–	–	–	–	**0.16**	[0.03–0.92]	0.041	**6.21**	[0.94–40.95]	0.058
Treatment 2												
BCS+RT	**1**			**1**			**1**			**1**		
Mastectomy, no RT	**1.52**	[0.75–3.11]	0.246	**1.05**	[0.47–2.38]	0.892	**1.97**	[0.75–5.19]	0.169	**1.21**	[0.41–3.56]	0.723
Mastectomy + RT	**1.18**	[0.52–2.68]	0.683	**2.21**	[1.18–4.14]	0.013	**1.26**	[0.41–3.82]	0.687	**3.44**	[1.44–8.22]	0.005
Others	**2.17**	[0.81–5.84]	0.124	–	–	–	**3.41**	[0.93–12.51]	0.065	–	–	–
Margins												
Negative	**1**			**1**			**1**			**1**		
Narrow	**1.42**	[0.59–3.42]	0.428	**0.84**	[0.3–2.37]	0.741	**2.37**	[0.64–8.72]	0.196	**1.33**	[0.3–5.98]	0.709
Positive	**2.43**	[0.99–5.99]	0.053	**2.88**	[1.28–6.49]	0.011	**1.14**	[0.15–8.79]	0.902	**5.39**	[1.7–17.06]	0.004
Chemotherapy												
No	**1**			**1**			**1**			**1**		
Yes	**0.65**	[0.32–1.32]	0.237	**1.02**	[0.43–2.44]	0.963	**1.10**	[0.35–3.42]	0.875	**0.38**	[0.11–1.31]	0.124
Hormonal treatment												
No	**1**			**1**			**1**			**1**		
Yes	**1.45**	[0.65–3.25]	0.365	**0.72**	[0.32–1.6]	0.416	**0.25**	[0.08–0.76]	0.014	**0.67**	[0.15–3.02]	0.602

When analyzing the cohort diagnosed during the period 2001–2012, three factors resulted significantly associated with a reduced risk of developing LR as a first event: period of diagnosis (HR: 0.83, 95% CI: 0.72–0.96); molecular subtype triple negative (HR: 0.16 as compared to Luminal A-like, 95% CI: 0.03–0.92); and treatment with hormonal therapy (HR: 0.25, 95% CI: 0.08–0.76). For DR as a first event, women in this cohort showed a lower risk if older than 40 years (HR: 0.41, 95% CI: 0.2-0-0.83) and higher risk if stage III at diagnosis (HR of stage III vs stage I: 4.99, 95% CI: 1.51–16.5); luminal B molecular type (HR: 3.17, 95% CI:1.18–8.55); had mastectomy plus RT as surgical strategy (HR: 3.44, 95% CI: 1.44–8.22); and positive margins after surgery (HR: 5.39, 95%CI: 1.70–17.06) (Table 2). Triple-negative tumors presented a HR of 6.21 compared to the reference Luminal A-like tumors, but the result did not reach statistical significance (*p*=0.054).

## Conclusion

In this cohort of women diagnosed with breast cancer at the age of 45 years or less followed a median of seven years, more than one-fourth experienced one of the study outcomes, including LR and DR, second breast cancer, or death as the first event. DR occurred only slightly more often than LR all along the follow-up period, and the risk remained important still at 10 and 15 years after the diagnosis. The timing and rate of LR and DR varied with age, tumor characteristics, and type of surgery. A lower risk of developing LR as first event was associated with older age on the overall cohort, and among women diagnosed more recently, with period of diagnosis, triple-negative molecular subtype, and use of hormonal therapy. Older age was also associated with a lower risk of developing DR, while more advanced stage, type of surgery strategy, positive margins after surgery, and molecular subtype of the tumor, for the subset of women for whom this could be defined, were all associated with a higher risk.

In the POSH longitudinal cohort study, Maishman et al. found that the frequency of LR among young breast cancer patients <40 years old at diagnosis was much lower than that of DR, at least within the first 10 years [20]. In our cohort, although DR were more frequent, the difference was very small, likely because local and regional recurrences were considered together.

Although the recurrences occurred all along the follow-up period, the majority of them was diagnosed during the first 5 years of follow-up. As showed for older breast cancer patients [21], the occurrence among young women seems to present two peaks, one during the first two years of follow-up and a second one at 5–7 years [22]. The higher risk of developing both LR and DR within the first two years of diagnosis was observed in our cohort among women younger than 40 years, women with stage III disease or with ER-negative cancers.

Younger age at diagnosis showed up as an independent factor associated with the development of LR as first event when using the whole cohort. This result is in line with most studies [3, 23]. Of note are the results of the analysis carried out on the sub-cohort of women diagnosed between 2001 and 2012 that showed a lower risk among women carrying triple-negative cancers to develop LR. This finding is consistent with those of a previous study of triple-negative breast cancer patients showing that these cancers more frequently develop a systemic recurrence, while a loco-regional recurrence occur more rarely [24, 25]. The concurrent much higher risk of DR, although not statistically significant, among triple-negative breast cancers observed in the multivariate model for the cohort of women diagnosed in 2001–2012 in the present study, strongly supports this argument.

In addition, our results showed a decreased risk of LR among women treated with hormonal therapy. While tamoxifen has been the standard of therapy for ER/PR-positive breast cancers regardless of age for many years, recently the use of aromatase inhibitors and the addition of Ovarian Function Suppression (OFS) have been included in the treatment of young women [26]. We could collect the type of treatment received for 491 out of the 776 women who had a hormonal treatment. Among them, 96% in the period 1995–2000 received tamoxifen, while in the period 2001–2012 80% received tamoxifen, 10% received an aromatase, alone or after tamoxifen, and 9% had also surgical or medical OFS.

As in other studies, we found that factors influencing the risk of DR were age, stage at diagnosis, and molecular subtype for the subset of women for whom this could be defined [7, 27].

Positive surgical margins after surgery were an independent factor increasing the risk of DR, in total agreement with the results of two recent systematic reviews and meta-analyses that showed that involved or close pathological margins are associated with an increased risk of local and distant recurrences both after breast-conserving surgery [28] and mastectomy [29].

Type of surgery strategy was also an important determinant of recurrence risk in several studies. Recently, Pederson et al. reported a lower risk of late breast cancer recurrences among patients who received BCS compared to those who received a mastectomy [27]. Similarly, Elder et al. in their study [30] and Ho-Huynh et al. in their systematic review of breast cancer outcomes in Australia [31] showed that women who underwent mastectomy were at increased risk of recurrence compared to women who underwent BCS. In our study, we also found that BCS had a similar or even protective effect toward the risk of DR in a multivariate model, confirming that young age is not a contraindication for BCS [32]. Post-mastectomy radiation therapy is indeed recommended for patients at high risk of recurrence, including those with involved axillary lymph nodes, positive resection margins, and T3–T4 tumors independent of nodal status [33]. The higher risk of DR confined to those patients who received both mastectomy and radiotherapy may indicate that these patients were indeed at higher risk and that our models do not fully adjust for all the factors that increase this risk.

This study has several strengths. It is a population-based cohort in Geneva of all women ≤45 years old diagnosed with breast cancer in a recent period with a follow-up to 20 years for death and recurrences. The study variables, including recurrence diagnosis, have been collected with high accuracy. Thanks to a strong and high-quality network acting in a restricted geographical area, we are confident that all available information is captured. Therefore, the assumption “no information, no recurrences” is trustworthy. This assumption was tested and validated for a 30 cases random sample for which active search was performed using all available sources, including direct contact with patients’ physicians. Furthermore, we used competing risk analysis that allowed to correctly estimate the absolute risk of recurrence in the population of young women, unbiased by other competing risk events.

The main limitation of the study is related to its observational nature. We had a lot of information available about each woman, and in the analysis of the determinants of recurrence, we included many of the variables considered important for the outcome of interest; however, we cannot assure to have eliminated all the possible bias and confounding. The small numbers of women in some subgroups did not allow for more detailed analysis. Our focus was on the first event and did not consider the risk of developing more than one event. Another limitation is the fact that not all the patients were classified according to surrogate molecular subtypings due to the lack of information on HER2 and Ki67 status before 2001. Finally, these findings are not directly applicable to current patients since standardized treatments offered now maybe different compared to those offered in the period under study.

Due to the increasing number of women being diagnosed with BC, also of young age, and the increasing number of long-term survivors, the estimation of the risk of recurrence and the identification of factors related to development of recurrence are of extreme importance. The prognosis of patients with recurrences, particularly distant recurrences, is less favorable and proper adjustments in the management of young patients are necessary to improve their outcomes.

Special attention is required to the type of surgery offered and the achievement of negative margins, as well as more aggressive adjuvant therapy in case of more advanced or poorly differentiated tumors, such as receptor-negative and/or HER2-positive tumors.

### Supplementary Information

Below is the link to the electronic supplementary material.

Supplementary file1 (DOCX 83 KB)

## Data Availability

The datasets used and analyzed during the current study are available from the corresponding author on reasonable request. In compliance with data protection regulations, data are stored at the Geneva Cancer Registry, Geneva, Switzerland, but are available from the corresponding author on reasonable request.

## Abbreviations

BC: Breast cancer
DR: Distant recurrence
LR: Loco-regional recurrence
HR: Hazard ratio
GCR: Geneva cancer registry
ER: Estrogen receptor
PR: Progesterone receptor
BCS: Breast-conserving surgery
