# Supplementary material for: Risk of first recurrence after treatment in a population-based cohort of young women with breast cancer
Source: Breast Cancer Res Treat. 2024 Apr 30;206(3):615–23. doi: 10.1007/s10549-024-07338-2 (PMC11208255; doi:10.1007/s10549-024-07338-2)
Supplement: Supplementary file 1 — Supplementary file1 (DOCX 83 KB) [file 10549_2024_7338_MOESM1_ESM.docx]

# Supplementary Figures

*Suppl. Figure 1. Cumulative incidence of recurrences of young women with breast cancer by age at 5 years. a) Loco-regional recurrences. b) Distant recurrences. Geneva 1995.-2012.*

*Suppl. Figure 2. Cumulative incidence of recurrences of young women with breast cancer by stage at 5 years. a) Loco-regional recurrences. b) Distant recurrences. Geneva 1995–2012.*

*Suppl. Figure 3. Cumulative incidence of recurrences of young women with breast cancer by grade at 5 years. a) Loco-regional recurrences. b) Distant recurrences. Geneva 1995–2012.*

*Suppl. Figure 4. Cumulative incidence of recurrences among young women with breast cancer by ER status at 5 years. a) Loco-regional recurrences. b) Distant recurrences. Geneva 1995–2012.*
